# The Effect of Strain and Rearing Medium on the Chemical Composition, Fatty Acid Profile and Carotenoid Content in Silkworm (*Bombyx mori*) Pupae

**DOI:** 10.3390/ani9030103

**Published:** 2019-03-20

**Authors:** Camilla Chieco, Lucia Morrone, Giampaolo Bertazza, Silvia Cappellozza, Alessio Saviane, Francesco Gai, Nicola Di Virgilio, Federica Rossi

**Affiliations:** 1 Institute of Biometeorology, National Research Council, via P. Gobetti 101, 40129 Bologna, Italy; l.morrone@ibimet.cnr.it (L.M.); g.bertazza@ibimet.cnr.it (G.B.); n.divirgilio@ibimet.cnr.it (N.D.V.); f.rossi@ibimet.cnr.it (F.R.); 2 Council for Agricultural Research and Economics, Research Centre for Agriculture and Environment—Sericulture Laboratory of Padua, Via Eulero, 6a—35143 Padova, Italy; silvia.cappellozza@crea.gov.it (S.C.); alessio.saviane@crea.gov.it (A.S.); 3 Institute of Sciences of Food Production- National Research Council, L.go P. Braccini 2, 10095 Grugliasco, Italy; francesco.gai@ispa.cnr.it

**Keywords:** insects, silkworm, rearing technology, alternative feed, protein source, fatty acid profile, carotenoids

## Abstract

**Simple Summary:**

The replacement of environmental-costly resources in food and feed production is now imperative. Insects are recognized to be an effective protein source alternative to fishmeal and soy for animal husbandry. The evaluation of their nutritive properties can offer important insights to determine their potential use as feed. This study compares the pupae body composition of two different silkworm strains: a conventional polyhybrid producing white cocoons and the Nistari with golden yellow cocoons, rich in carotenoids, fed either on fresh mulberry leaves or an artificial diet. The results establish that feeding substrate composition strongly influences the fat and protein content of silkworm pupae. The feeding substrate also positively influences the pupae’ n-3/n-6 ratio while the carotenoid content is exclusively determined by the strain.

**Abstract:**

The overexploitation of fishmeal and soy for the feedstuff industry has provided an opportunity to employ insects as an unconventional and more environmental friendly protein source. The evaluation of the nutritive properties of different insect species has consequently become a priority. The present study compares the pupal nutritive composition of two silkworm strains (White Cocoon Polyhybrid and Golden Yellow Cocoon Nistari) fed on two different rearing media (fresh mulberry leaves and a commercial artificial diet). Our results provide evidence that the composition of the feeding substrate strongly influences the fat and protein content of silkworm pupae. The two tested strains had higher fat and lower protein contents when fed with silkworm natural food (mulberry leaves) with respect to the commercial artificial diet. The analysis also showed that the n3/n6 ratio was affected almost exclusively by the feed substrate factor. On the contrary, the carotenoid content in pupae was specifically determined by the strain. The study identifies the interesting opportunity offered by silkworm pupae, which are usually a waste product of the silk-reeling process, to be used as alternative animal protein sources in a fully-closed circular production.

## 1. Introduction

Animal husbandry, due to the long feed chain, is highly demanding in terms of resources and is a remarkable source of greenhouse gases (GHG), accounting for almost 12% of global, 19% of anthropogenic, and 36% of agricultural emissions [1]. Along the feed chain, a large portion of GHG is related to feed field production, especially N_2_O emissions from soil [2]. An environmental priority is to identify and introduce new environmental-friendly and minimum-waste feed resources, especially in terms of protein content. Fishmeal and fish-oil are among the most exploited sources of proteins and fatty acids for feeding livestock and aquaculture [3]. Approximately 25% of these components are by-products of human fish consumption, while the remaining components come from fish purpose-caught in the open sea [4]. This causes an over-exploitation of pelagic resources due to incidental catches of juvenile forms of commercial species. Fishmeal and fish-oil are becoming scarce and expensive, and their quality is not constant because their supply depends on the fish abundance and reproduction rate, which is in turn affected by natural phenomena, such as extreme El Niño events due to climate change [5].

Replacing fish-based protein with less commercial and environmentally expensive protein sources has become a common practice in the past decades [6]. Soybean meal is the protein supplement most frequently included in diets for animals, mainly for poultry and pig [7]. However, soybean contains some antigenic proteins and oligosaccharides, such as raffinose and stachyose, which are not digested in monogastric intestines and necessitate undesirable and expensive ethanol treatment to be inactivated or removed [8]. Soybean meal is also scarce in sulphur-containing amino acids (methionine and cysteine) [9] and it contains trypsin inhibitors that reduce protein digestibility, especially in salmon [10]. In addition, massive soybean cultivation for animal feedstuff causes concern due to increasing conversion of forest to agricultural areas and related higher consumption of water, pesticides, and fertilizers [11].

Replacing environmentally-expensive proteins for feedstuff formulations, and completing animal diets with less impactful, highly digestible, and nutrient balanced sources is now a recognized and most urgent need.

The potential of insects as an alternative protein source for animal feed is widely recognized [9,12]. In particular, several studies have focused on the nutritive properties of silkworm (*Bombyx mori*, Linnaeus, 1758) since the dried pupae left out as wastes from the silk-reeling process may provide important and cheaper complementary protein sources [13].

Fish and broiler dietary meal has been integrated with various levels of silkworm pupae without adverse effects on animal growth, performance, and survival parameters [12,13,14]. Silkworm pupae are recognized to be rich in proteins and their amino acid composition is comparable to that of fish meal [15,16]. Pupae are also sources of essential fatty acids (EFA), especially polyunsaturated (PUFA), and, differently than other insects [9], have a higher n-3 to n-6 fatty acids ratio [17,18].

However, limited attention has so far been devoted to the nutritive properties of various silkworm mutants, in particular, coloured cocoon strain mutants, including yellow and golden yellow. Despite their lower silk productivity, such less explored strains retain some interesting biological characteristics, such as higher resistance to diseases and the ability to absorb carotenoids and flavonoids from the feed substrates [19]. Such pigments enhance protection against UV radiation through antioxidants [20] and serve the feedstuff industry (particularly fish farming and poultry industry) by imparting desired coloration to flesh [21] and eggs [22].

In addition, the information about the silkworm’s nutritional properties refers to pupae reared on silkworm natural food, including the fresh leaves of the mulberry (*Morus alba* L.), while there is no available knowledge on the nutritive content of pupae reared on an artificial diet. The use of artificial diets for silkworm rearing is a recognized method that enables a stable production [23] and has the advantage of being able to be functionalized with additives (carotenoids, flavonoids).

The nutritional value of the pupae of a coloured silkworm mutant, the Nistari strain with Golden Yellow cocoons (GN), has been assessed here in comparison with a conventional Polyhybrid strain producing white cocoons (WP) as a first criterion to verify the potential added value resulting from using an alternative silkworm strain.

Additionally, considering that the nutrient quality of insects is highly dependent on the feed composition [12,24], we report on the pupal nutritive composition derived by feeding larvae on two different substrates, fresh mulberry leaves and a commercial artificial diet, to determine how the alimentary substrate affects the body composition of the insect.

## 2. Materials and Methods

### 2.1. Silkworm Rearing

Silkworm larvae were reared at the Sericulture Laboratory of Padua (CREA-AA) that preserves about 200 accessions, including geographical strains, mutants, and pure lines of *B. mori*. Two strains, Golden Yellow Nistari (GN) and White Polyhybrid (WP), which was obtained by the cross of Japanese and Chinese pure lines (four-way hybrid: 118 × 129–120 × 125), were studied. The two strains represent the opposite conditions: Nistari is very similar to the wild ancestor of *B. mori*, having a coloured cocoon, where pigments contained in the ingested mulberry leaf are stored through digestion in the midgut and transported in the haemolimph. The white cocoon hybrid is a cross of mutant strains widely reared on a massive scale.

Rearing was carried out at the temperature of 25 ± 1 °C and photoperiod of 16 h dark: 8 h light. Relative humidity (RH) varied depending on the instar; first instar: RH 85 ± 5%, second instar: 80 ± 5%, third instar: 75 ± 5%, fourth instar: 70 ± 5%, fifth instar: 65 ± 5%. RH was lowered to 60 ± 5% during the moulting period to permit diet desiccation. Larvae of both the strains were fed with two feeding substrates: fresh *M. alba* leaves of the Florio cultivar and a commercial artificial diet according to [25].

### 2.2. Samples Collection

Fresh mulberry leaves (ML) were randomly sampled from plants of the Florio cultivar maintained at the experimental field of CREA-AA. Fresh leaves were flash-frozen in liquid nitrogen and stored at −80 °C.

The commercial artificial diet (AD) (100 g of dry diet in powder added to 260 mL of water, mixed and sterilized in an autoclave for 40 min at 105 °C) was also stored at −80 °C. Before the analysis, both feeding substrates were lyophilized.

100 g of freshly spun cocoons were randomly collected from the different rearing thesis, oven dried for 3 days at 60 °C, and the pupae were extracted and maintained at −80 °C until analysis. Frozen pupae were milled to a fine powder (pupa meal) using a laboratory scale grinder (IKA, Staufen, Germany).

### 2.3. Chemical Characterization

Moisture, crude protein, crude fat, fatty acid profile, and carotenoids were quantified for the feeding substrates and silkworm pupae. For the feeding substrate, the total free sugar was also measured. Each analysis was carried out in triplicate and data are expressed as a percentage of the dry matter.

For moisture, 10 g of feeding substrates and 5 g of pupae of different theses were dried to a constant weight in an oven at 60 °C for 3 days.

The total nitrogen content was determined in 10 g of feeding substrates and in 5 g of pupa meal of different treatments using a nitrogen analyzer (Rapid N III, ElementarAnalysensysteme GmbH, Germany) according to the Dumas method and the crude protein was estimated as N × 6.25 [26].

For free sugar determination, 1 g of freeze-dried samples of substrates was extracted twice with 20 mL of a solution of imidazole buffer 0.05 M, pH 7: ethanol (1:1; v:v), for 12 h. 20 mg of b phenyl D-glucopyranoside were used as internal standard. After centrifugation (9000 rpm for 6 min), the supernatants were combined, and 4 mL of extract were washed twice with 4 mL of chloroform. After purification, the extract was dried by air flow. The dry residue was oximated with 400 µL of a solution of hydroxylamine/pyridine (1:20; p:v) and then silanized with 400 µL of a pyridine:hexamethyldisilazane:trimethylchlorosilane (1:4:1; v:v:v). 0.5 mL derivatized sample was injected in a gas chromatograph (Varian CP 3800) equipped with an injector splitter, a flame ionization detector, and a capillary column (HP1 30 m, 0.25 mm ID df 0.25 mm Agilent). The retention times of standards of the main sugars and organic acids present in the samples were used for qualitative determination. The quantification of each compound was performed using the internal standard calculation method.

For crude fat quantification in the feeding substrates, the Bligh and Dyer method [27] was used. 10 g of lyophilized feeding substrate was homogenized with 60 mL of chloroform-methanol (1:2; *v*/*v*) solution. After adding 20 mL of chloroform and 40 mL of deionized water, the mixture was homogenized, filtered under vacuum, and the organic phase was collected and evaporated to dryness in a rotary evaporator; lipids were gravimetrically determined.

Crude fat content in pupae was determined according to Folch’s method [28]. 1.5 g of pupae meals was homogenized with 30 mL of chloroform/methanol (2:1; *v*/*v*) solution, the mixture was centrifuged, and the organic phase collected in a test tube. The extraction was repeated; the two organic phases were filtered and combined. A weak salt solution was added at a final ratio of 8:4:3 chloroform/methanol/water. After centrifugation, the chloroform lipid-containing layer was collected, evaporated, and gravimetrically determined.

After the total lipid determination, all samples were re-suspended in 4 mL of heptane for fatty acid profile detection according to [29]. Lipid extract (1 mL) was macerated in 400 µL of a 0.1 M KOH solution in methanol and vortexed for 1 min. The supernatant (0.5 µL) was injected in the Varian CP-3800 gas chromatograph equipped with a 30 m long, ID 0.32 mm, df 0.25 µm Mega-10 high polarity column (Mega, Milan, Italy). The injector and detector temperature was 260 °C and the analytes were separated by the following temperature gradient: Initially, T° 140 °C for 1 min, then from 140 °C to 200 °C with an increment of 5 °C/min, subsequently from 200 °C to 260 °C with an increase of 10 °C/min, and finally 260 °C for 3 min.

Solvents for carotenoid detection were chosen according to [30] and [31]. For analysis, 1 g of sample was extracted at −20 °C by alternating for three times 5 mL of ethanol and twice 5 mL of tetrahydrofuran for 24 h. Each step was performed for a total of 5 days to obtain a clear pellet. Combined supernatants were centrifuged at 14,000 rpm for 5 min and 20 µL of this solution was injected in a HPLC system equipped with a dual piston solvent delivery pump LC-10ADvp (Shimadzu, Kyoto, Japan), a low pressure quaternary gradient mixer FCV-10ALvp (Shimadzu, Kyoto, Japan), a thermostated column compartment CTO-10ASvp (Shimadzu, Kyoto, Japan), a vacuum solvent delivery degassers Gastorr 154 (Flom; San Diego, CA, USA), a photodiode array detector UV6000LP (Thermoquest; Waltham, MA, USA), and a chromatographic column Adsorbosphere UHS C18, 150 × 4.6 mm, particle size 5 µm (Alltech; Lokeren, Belgium). According to [32], samples were eluted at a flow rate of 1.4 mL/min by 2 mobile phases: Solution A tris buffer 0.01M pH 7.5—acetonitrile (60-40, v-v), solution B acetonitrile—dioxane (65–35, v-v). For elution, a linear gradient was used: T0 min 35% sol. A and 65% sol. B; T50 min 0% sol. A and 100% sol. B; then, an isocratic gradient was used: From T50 min to T65 min by sol. B 100%

### 2.4. Statistical Analysis

The Student’s *t*-test was used to detect the differences in means between the two feeding substrates. For pupae, the effect on the chemical composition of the strains and feeding substrates as well as the interaction between these two factors were evaluated by two-way ANOVA followed by a Tukey’s honestly significant difference (HSD) test to establish significant differences at a 5% level. A principal component analysis (PCA) was performed on the pupae datasets to represent the correlation between the data analysis from the diets and pupae. Statistical analyses were performed using Microsoft^®^ Excel 2007/XLSTAT^©^ (Version 2009.3.02, Addinsoft, Inc., Brooklyn, NY, USA).

## 3. Results

### 3.1. Chemical Composition of Feeding Substrates

The chemical composition of the two feeding substrates is reported in Table 1. Fresh mulberry leaves’ moisture content was 67.8%, against 73.2% of the diet. The two feeding substrates had similar crude fat contents, while they were statistically different in the total protein and total free sugar amount, as Florio leaves contained almost 40% less crude protein and twice as much sugar content than the artificial diet.

The two feeding substrates differed mainly for the content of the linoleic acid (C18:2) and linolenic acid (C18:3). In both substrates, in fact, polyunsaturated fatty acid (PUFA) accounted for more than 60% of the total fat, but in fresh leaves, the highest percentage was constituted by C18:3, while the artificial diet had a higher amount of C18:2.

Carotenoids in the mulberry leaves were higher for lutein and β-carotene. Other carotenoids in the leaves were neoxanthin, violaxanthin, and antheraxanthin. Only lutein and β-carotene were found in the artificial diet, with a total pigment content lower than in the fresh leaves.

### 3.2. Chemical Composition of Silkworm Pupae

Data on the pupal proximate composition are reported in Table 2. Pupae of the Nistari strain had a higher moisture percentage than the Polyhybrid regardless of the artificial diet on which larvae matured. Both strains had a higher fat content when fed on fresh mulberry leaves; White Polyhybrid reached a total fat content of 33.3%, which decreased by 10% when fed on the diet. Likewise, Golden Nistari fed on fresh leaves showed a total fat content of 30.6%, which decreased by 5% in the same strain fed on the artificial diet.

Polyhybrid and Nistari fed on mulberry leaves had, respectively, a protein content of 53.1%, and 56.4%. Feeding larvae on the artificial diet led to an increase of these percentages (between 6% and 8%).

The fatty acid profiles of pupae are reported in Table 3. Both strains had the highest amounts of saturated fatty acids (SFA), consisting mostly of palmitic (16:0) and stearic acids (C18:0) and monounsaturated fatty acids (MUFA), represented by palmitoleic (C16:1) and oleic acid (C18:1), when fed an artificial diet. Oleic acid was the most representative fatty acid in silkworm pupae, reaching the highest percentage in the Nistari strain fed with an artificial diet.

Regarding the PUFA content, consisting of linoleic (C18:2) and linolenic acid (C18:3), larvae grown on the artificial diet showed similar amounts, while, when fed with fresh mulberry leaves, the pupae increased the C18:3 content at the expense of C18:2.

In both the analysed strains, lutein was the most abundant carotenoid, followed by β-carotene, the only carotenoids present (Table 4). The Golden Nistari strain always had a higher total carotenoid content with respect to the White Polyhybrid regardless of the rearing substrate. Considering the feeding substrate, pupae fed on the artificial diet had a lower carotenoid content when compared with those fed with fresh leaves.

### 3.3. Multivariate Data Analysis

**Figure 1:**
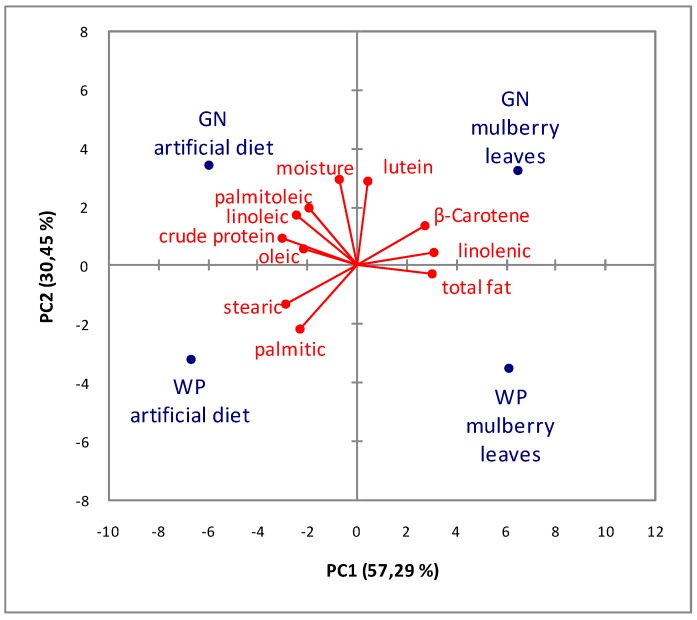
PCA analysis of the chemical composition of the silkworm pupae of Polyhybrid (WP) and Nistari (GN) strains fed an artificial diet (AD) and fresh mulberry leaves (ML).

To visualize the chemical divergences of the two strains determined by their growing substrates, a PCA was performed using the data of the silkworm chemical composition. The statistical model of PCA proposed three significant components for a total variance of 11. The first two components explain about 88% of the variance, whereas the variance explained by PC3 was only 12%, so only the first two PC were considered. From the results reported in the biplot of Figure 1, we can deduce that 57% of the variance is associated to the rearing medium: on PC1, all strains fed on the ML diet (right quadrant) had positive scores, whereas strains fed with the AD (left quadrant) had negative scores. PC2, which explained 30% of the variance, was dominated by the type of strain: Golden Nistari had positive scores, and WP had negative scores. Specifically, silkworm pupae of the White Polyhybrid strain fed an artificial diet were characterized by a higher saturated fatty acid content with respect to the other groups; the same strain fed on fresh mulberry leaves was characterized by a larger total fat content with respect to the other samples.

Compared to other samples, the Golden Nistari strain fed an artificial diet was characterized by a higher crude protein content and by a larger oleic, linoleic, and palmitoleic acid amount; the same strain grown on fresh mulberry leaves was instead characterized by a higher carotenoid component and by a greater linolenic (n-3) amount with respect to the other samples.

## 4. Discussion

The chemometric analysis confirmed the differences between the two strains and highlighted the influence of the feeding substrate on the pupa composition. From their positioning on the PCA, it was clear that the two strains differed from each other and that their chemical divergence was accentuated by the type of growing substrate. These results lead us to suppose that the chemical composition of pupae is influenced primarily by the diet, and secondly by the strain.

Pupal moisture was mainly affected by the strain and was less influenced by the feeding substrates and by the interaction of the two factors. In fact, despite the pronounced difference of the moisture between the two alimentary substrates, Nistari pupae resulted in a higher moisture percentage with respect to the Polyhybrid pupae, regardless of the diet.

On the contrary, consistent with [9], feeding substrates strongly influenced the fat and protein content of silkworm pupae.

Despite the similar fat content of the two feeds, both strains had a higher fat content when fed fresh leaves, a substrate characterized by a major sugar content. Likewise, the protein content increased when the strains were fed an artificial diet, reflecting the major protein content of this substrate.

Moreover, the artificial diet decreased differences between the two strains; in fact, larvae of the two strains fed with fresh leaves gave place to a pupal composition significantly different (for a probability level >95%) between the strains, whereas when fed an artificial diet, the pupal composition was similar in both the strains.

The fatty acid profile of pupae fed with mulberry leaves was in line with those reported in the literature for silkworm pupae fed in the same way [16,18]. The principal factor that influenced the fatty acid composition of pupae was the feeding substrate, with the exceptions of C16:0 (palmitic acid) and C18:1 (oleic acid), which were, respectively, mostly influenced by the strain and by the interaction between strain and feeding substrate. C18:3 was the fatty acid most influenced by the substrate composition and its content in pupae reflected its amount in the substrate. The diet based on fresh leaves induced a higher PUFA content in both strains, with percentages in line with those reported by many authors [33]. High levels of PUFAs are typical in insects, especially n-6 [34], while the greater part of the insects considered useful as fish meal is scarce in n-3 [9]. The analysis of variance showed that the ratio of n-3 to n-6 fatty acid (n3/n6) was almost exclusively affected by the feed substrate factor. In the fat of silkworm pupae grown on fresh leaves, linolenic acid (n-3) was more than three times more represented with respect to linoleic acid (n-6) for White Polyhybrid and 2.8 times higher for the Golden Nistari. When larvae were fed an artificial diet, instead, the ratio of n3/n6 was approximately 1:1 in the pupae.

These results are consistent with numerous reports affirming that the fatty acid profiles of insects reflect the composition of their food [9], thus reinforcing the fact that the nutritive value of insect meal could be amplified by acting on the alimentary substrate [29]. Moreover, although the fatty acid content of silkworm pupae is different from that of fish meal, which is especially rich in long chain n-3 polyunsatured fatty acids, the high content of C 18:3, a precursor of the n-3 series, makes these insects good candidates as oil sources in the formulation of feedstuff.

Carotenoids comprise more than 600 compounds that consist of hydrophobic pigments, affecting insect vision and other important functions, such as coloration, camouflage, and sexual attraction. Animals, including insects, are not able to synthesize these molecules, thus they must be acquired from dietary sources [35]. Yellow and green silkworm races result in coloured cocoons since they have specific hemolymph lipophorins that are able to transport pigments from the midgut lumen to the silk gland. Some white cocoon silkworm mutants (+C) assimilate carotenoids from food, but, unlike yellow strains, are not able to store pigments in cocoons, as they are deficient in one or more hemolymph lipophorins necessary for their translocation [36].

Artificial diet preparation involves subsequent thermal treatments (oven and autoclave) that affect the carotenoid content [37]. Nevertheless, the almost seven times higher carotenoid content of fresh mulberry leaves with respect to the artificial diet did not determine a corresponding increase of the pigment content in the pupae. Based on our experiments and from PCA, we can infer that the total carotenoid content was mainly influenced by the strain while the substrate composition accounted for less than 20% of the variation. Strain especially influenced the lutein content, while the feed substrate did not show significance as a variability source. In contrast, variation in β-carotene was mainly due to substrate composition, whereas strains accounted for 20.6% of the variation.

Carotenoids have a double function in feedstuff formulation. Because of their ability to sequester free radicals formed during oxidative processes, they impart stability to the lipid content, preventing or reducing deteriorative processes in stored feed and prolonging safe storage. At the same time, carotenoids colour many important fish and shellfish products, and broilers’ skin and eggs. A range of synthetic and natural carotenoids are available for the aquaculture and poultry industry for pigmenting flesh and eggs [22]. In farmed salmonids, astaxanthin and canthaxanthin are the principal carotenoid supplements for flesh coloration [21], while lutein rich Aztec marigold flowers (*Tagetes erecta*) are the most widely used sources of concentrated xanthophylls to pigment broiler skin and eggs [38]. Natural pigments incorporated into the pupae of Golden Yellow Nistari silkworms could become a cost-effective solution for the formulation of less expensive feeds, considering that adding pigments, especially from natural sources, entails a cost increase of 8% to 10% to the diet cost [38].

## 5. Conclusions

This investigation has shown that the Golden Yellow Nistari strain has particular nutritional properties that could make it an interesting alternative to conventional animal protein sources. This insect represents a natural source of carotenoids, especially when fed mulberry leaves. Such natural food also favours the accumulation of beneficial n-3 fatty acids in both the silkworm races. Considering that the scarcity of n-3 in respect to n-6 and the lack of EPA and DHA are the most important drawbacks in the use of insects as fish feed [9], the possibility of positively influencing the silkworm n-3/n-6 ratio using an appropriate feeding substrate may represent an added value in the use of silkworm oil in feed formulation. Even though the protein content was maximum when the insect was fed an artificial diet, using fresh mulberry leaves as feeding substrates gives the silkworm pupae a protein content comparable to that of soybean [9], which is conventionally used as protein source, especially for poultry.

An increasing consciousness about the opportunities offered at various levels by insects to different branches of emerging bioeconomical issues is largely arising. Besides other potential utilizations of several species for human nutrition, new perspectives have been opened, for example, to utilize silkworm by-products as the fibroin for advanced hi-tech industrial applications [39,40]. Also, in that case, coloured strains demonstrated their remarkable value. The study carried out here identifies the interesting opportunity for a fully-closed circular *B. mori.* production, including the use of dried pupae in a protein production chain.

## Figures and Tables

**Table 1:** Compositional analysis of the artificial diet (AD) and mulberry leaves of Florio cultivar (ML).

Chemical Component	AD	ML	*p*-Value
**Moisture (%)**	73.2 ± 0.2	67.8 ± 1.1	0.001
**Crude fat (%)**	3.2 ± 0.1	4.0 ± 0.7	0.096
**Crude Protein (%)**	21.1 ± 0.6	12.4 ± 0.3	0.003
**Total free sugar (mg/g)**	59.4 ±2.6	104 ± 2	<0.0001
**C16:0 (%)**	23.0 ± 0.5	20.1 ± 0.4	0.001
**C16:1 (%)**	1.3 ± 0.1	1.4 ± 0.1	0.172
**C18:0 (%)**	4.2 ± 0.1	4.3 ± 0.1	0.458
**C18:1 (%)**	10.3 ± 0.3	9.5 ± 0.3	0.033
**C18:2 (n-6) (%)**	33.6 ± 0.2	27.5 ± 0.3	<0.0001
**C18:3 (n-3) (%)**	26.8 ± 0.3	36.5 ± 0.5	<0.0001
**SFA (%)**	27.2 ±0.4	24.4 ±0.5	0.001
**MUFA (%)**	11.6 ±0.3	10.9 ± 0.3	0.037
**PUFA (%)**	60.4 ± 0.1	64.0 ± 0.6	0.001
**n3/n6**	0.8 ± 0.1	1.3 ± 0.1	<0.0001
**Neoxanthin (ug/g)**	nd	38.8 ± 5.6	
**Violaxanthin (ug/g)**	nd	51.9 ± 2.5	
**Antheraxanthin (ug/g)**	nd	26.4 ± 1.0	
**Lutein (ug/g)**	47.0 ± 1.9	197 ± 9	<0.0001
**β-Carotene (ug/g)**	21.6 ± 1.1	148 ± 9	<0.0001
**Tot. carotenoids (ug/g)**	68.6 ± 2.8	462 ± 25	<0.0001

Data are expressed as mean ± standard deviation. *p* < 0.05; nd, not detected.

**Table 2:** Moisture, crude fat content, and crude protein content of the pupae of White Polyhybrid (WP) and Golden Nistari (GN) strains fed with an artificial diet (AD) and mulberry leaves (ML).

Rearing Medium	Strain	Moisture (%)	Crude Fat (%)	Crude Protein (%)
**AD**	**WP**	73.0 ^a,b^ ± 1.0	23.3 ^c^ ± 0.1	61.9 ^a^ ± 1.5
	**GN**	76.8 ^a^ ± 0.3	25.1 ^c^ ± 1.1	62.7 ^a^ ± 0.1
**ML**	**WP**	73.0 ^b^ ± 0.7	33.3 ^a^ ± 0.8	53.1 ^c^ ± 0.4
	**GN**	75.1 ^a,b^ ± 0.4	30.7 ^b^ ± 0.9	56.4 ^b^ ± 0.4
Strain		85.4 ***	0.3 ns	6.9 *
FeedSubstr		7.5 **	92.2 ***	90.4 ***
StrainX FeedSubstr		7.2 *	7.6 **	2.7 ns
*p*-value		<0.0001	<0.0001	0.001

Data are expressed as mean ± standard deviation. Different letters (a,b,c,d) in the same column show significant differences among different mean values according to Tukey HSD (*p* < 5%); variability expressed as a percent of the total sum of the squares; * *p* < 0.05, ** *p* < 0.01, or *** *p* < 0.001 levels, ns: not significant.

**Table 3:** Fatty acid composition (% of total fatty acids) of silkworm pupae of White Polyhybrid (WP) and Golden Nistari (GN) strains fed an artificial diet (AD) and mulberry leaves (ML). SFA—saturated fatty acid; MUFA—monounsatured fatty acid; PUFA—polyunsaturated fatty acid; n3/n6—ratio of n-3 to n-6 fatty acid.

Rearing Medium	Strain	C16:0	C16:1	C18:0	C18:1	C18:2 (n-6)	C18:3 (n-3)	SFA	MUFA	PUFA	n3/n6
**AD**	**WP**	29.2 ^a^ ± 0.4	1.5 ^a^ ± 0.0	10.9 ^a^ ± 0.1	35.1 ^b^ ± 0.3	11.4 ^a^ ± 0.5	11.7 ^c^ ± 0.3	40.1 ^a^ ± 0.5	36.6 ^b^± 0.4	23.1 ^c^ ± 0.8	1.0 ^c^ ± 0.0
	**GN**	25.1 ^b^ ± 0.3	1.5 ^a^ ± 0.0	9.3 ^b^ ± 0.2	39.8 ^a^ ± 0.3	11.8 ^a^ ± 0.1	12.3 ^c^ ± 0.2	34.3 ^b^ ± 0.5	41.3 ^a^ ± 0.3	24.1 ^c^ ± 0.3	1.0 ^c^ ± 0.0
**ML**	**WP**	25.2 ^b^ ± 0.7	0.8 ^b^ ± 0.1	7.2 ^c^ ± 0.2	35.2 ^b^ ± 0.3	7.1 ^c^ ± 0.4	24.3 ^b^ ± 0.2	32.4 ^b^ ± 0.8	36.0 ^b^± 0.3	31.4 ^b^ ± 0.7	3.4 ^a^ ± 0.2
	**GN**	21.6 ^c^ ± 0.4	1.4 ^a^ ± 0.0	4.8 ^d^ ± 0.1	32.3 ^c^ ± 0.1	10.4 ^b^ ± 0.2	29.3 ^a^ ± 0.2	26.3 ^b^ ±0.5	33.8 ^c^± 0.2	39.7 ^a^± 0.5	2.8 ^b^ ± 0.1
Strain		51.3 ***	35.0 ***	19.9 ***	2.7 ***	24.8 ***	3.3 ***	35.3 ***	4.7 ***	12.2 ***	1.9 ***
FeedSubstr		48.4 ***	37.1 ***	79.4 ***	47.1 ***	60.5 ***	94.5 ***	64.7 ***	54.4 ***	80.2 ***	96.0 ***
StrainX FeedSubstr		0.3 ns	27.9 ***	0.8 **	50.2 ***	14.7 ***	2.2 ***	0.0 ns	40.9 ***	7.7 ***	2.0 ***
*p*-value		<0.0001	<0.0001	<0.0001	<0.0001	<0.0001	<0.0001	<0.0001	<0.0001	<0.0001	<0.0001

Different letters (a,b,c,d) in the same column show the significant differences among average values of HSD (*p* < 5%); variability expressed as a percent of the total sum of the squares; ** *p* < 0.01, or *** *p* < 0.001 levels, ns: not significant.

**Table 4:** Carotenoids content (µg/gr pupae) of White Polyhybrid (WP) and Golden Nistari (GN) strains fed an artificial diet (AD) and mulberry leaves (ML).

Rearing Medium	Strain	Lutein	β-Carotene	Total
**AD**	**WP**	2.4 ^d^ ± 0.1	0.6 ^d^ ± 0.2	3.0 ± 0.1
	**GN**	27.0 ^a^ ± 0.1	2.2 ^c^ ± 0.2	29.2 ± 0.2
**ML**	**WP**	11.1 ^c^ ± 0.3	6.0 ^b^ ± 0.3	17.1 ± 0.5
	**GN**	21.7 ± 0.2	13.3 ^a^ ± 0.4	35.0 ± 0.5
Strain		85.6 ***	20.6 ***	80.6 ***
FeedSubstr		0.8 ***	71.1 ***	16.5 ***
StrainX FeedSubstr		13.6 ***	8.3 ***	2.9 ***
*p*-value		<0.0001	<0.0001	<0.0001

Data are expressed as mean ± standard deviation. Different letters (a,b,c,d) in the same column show significant differences among different mean values according to Tukey HSD (*p* < 5%); variability expressed as percent of the total sum of the squares; * *p* < 0.05, ** *p* < 0.01, or *** *p* < 0.001 levels, ns: not significant.
